# Extracellular Vesicles and Cell–Cell Communication: New Insights and New Therapeutic Strategies Not Only in Oncology

**DOI:** 10.3390/ijms21124331

**Published:** 2020-06-18

**Authors:** Frank Gieseler, Fanny Ender

**Affiliations:** Experimental Oncology, University Hospital and Medical School (UKSH), University of Luebeck, Ratzeburger Allee 160, 23538 Luebeck, Germany; fanny.ender@uksh.de

The discovery that tumors are not separated from systemic regulatory mechanisms of the body but are rather integrated into them, and even use these mechanisms for their own purposes, was one of the most important findings in tumor biology of the last few years. In fact, the elucidation of the signaling pathways necessary for tumor cell proliferation and metastatic spreading, and the discovery that they can be blocked by molecularly targeted drugs, has led to completely new and successful therapeutic approaches in cancer therapy [1]. Tumor–host interactions can be systemic, e.g., by hormones and growth factors [2], but also local within the so-called “microenvironment” [3]. Determining the contribution of the microenvironment to cancer progression is challenging because it cannot be examined directly within the body on a cellular level and ex vivo models are always incomplete. Nevertheless, advances in our understanding of tumor biology are promising with regards to the development of new and alternative therapeutic strategies. It is generally assumed that the survival of tumors depends to a large extent on their successful integration into the microenvironment and their active adaptation with both positive survival signaling and the suppression of host-anti-tumor mechanisms, enabling their escape from immune surveillance [3,4]. One example of the successful therapeutic implementation of this knowledge on the escape mechanisms of tumor cells from the cellular immune system is targeting the PD1/PD-L1 axis using monoclonal antibodies. This targeted therapeutic approach has resulted in impressive success in tumors that are primarily difficult to treat, such as non-small cell lung carcinoma, glioma and melanoma [5,6,7].

A newly discovered molecular pathway for cell–cell interactions is represented by extracellular vesicles (EV) that are released by tumor cells as well as host cells. EVs are able to provide molecular signals between cells of different origin [8,9]. Examples of two principal mechanisms are the intercellular transfer of the oncogenic receptor EGFRvIII by EVs from tumor cells [10] and the induction of multidrug resistance (MDR) through microRNA transfer [11]. We are just beginning to understand the possible roles of EVs in cell–cell communication, especially in the malignant microenvironment [12]. One of the reasons for our lack of knowledge is the absence of the technology required to effectively separate different EV subtypes, especially exosomes and ectosomes (microvesicles). The problem of including EVs of undefined biological origin in experiments is that it regularly leads to confusion regarding the results. In order to compare the results of different working groups with each other, it is essential to be aware of the materials that the experiments are carried out with [13].

Four years ago, Gardiner et al. conducted a worldwide survey on the techniques used for the isolation and characterization of EVs and found differential ultracentrifugation to be the most commonly used primary technique for their separation and concentration [14]. Reflected by the research paper submitted to this Special Issue, differential centrifugation is still most commonly used for EV purification (four out of five submissions, 80%) [15,16,17,18]. However, methodological protocols still vary widely. Therefore, a consensus on accurate purification protocols within the scientific community is urgently needed. Besides centrifugation, one research group used the ExoQuick-TC^TM^ kit [19], ostensibly for the exclusive purification of exosomes. However, according to the product data sheet, exosomes as well as microvesicles between 30 and 200 nm in size can be precipitated from various fluids. Thus, this method, like differential centrifugation, isolates a mixture of different EV subpopulations; consequently, the resulting EV-mediated biological effects cannot be attributed to a specific EV subpopulation.

For the characterization of EVs, the International Society for Extracellular Vesicles (ISEV) suggests evaluating at least one protein from categories one to three (1. Transmembrane or glycosylphosphatidylinositol (GPI)-anchored proteins (GPI-APs) attached to the external leaflet of the plasma membrane and/or endosomes; 2. Cytosolic proteins recovered in EVs; 3. Major components of non-EV co-isolated structures) in any EV preparation. Furthermore, the analysis of proteins from categories four to five (4. Transmembrane, lipid-bound and soluble proteins located in intracellular compartments other than endosomes and not anchored to the plasma membrane; 5. Secreted proteins recovered with EVs) is recommended for studies that focus on one or more EV subtypes or that have identified a functional soluble factor in EVs [20]. Regarding the research contributions to this Special Issue, the ISEV guidelines are widely accepted, as scientists have implemented commonly used markers, such as tetraspanins (i.e., CD9, CD63, CD81), the tumor susceptibility gene 101 protein (TSG101), ALG-2 interacting protein X (ALIX), heat shock protein 90 (HSP90) and glyceraldehyde-3-phosphate dehydrogenase (GAPDH) for the characterization of their respective EV population of interest [15,16,17,18].

This Special Issue focuses on the decisive role of EVs as mediators in cell–cell communication, not only in health and disease but also for therapeutic approaches. Thus, the potential contribution of EVs to immune tolerance during pregnancy, the capability of dermis-derived EVs to influence skin senescence and the role of EVs in learning and memory are addressed in [16,17] and [21], respectively. The involvement of EVs in neuropathology is discussed with a particular focus on Alzheimer’s and Parkinson’s disease in [21]. In the context of cancer, the current knowledge on the role of EVs in oral squamous cell carcinoma/oral potentially malignant disorders and epithelial–mesenchymal transition is summarized by Yap et al. [22] and Kletukhina and colleagues [23], respectively. Furthermore, one contribution shows experimentally that HIV and amyloid β profoundly remodel the proteome of brain endothelial EVs, which in turn contributes to the pathology of HIV-infected brains [19]. The suitability of cardiac EVs for therapeutic applications is reflected by their pro-angiogenic effects on endothelial cells, as investigated by Beez and colleagues [15]. In addition, the use of EVs as a potential therapy for acute and chronic lung disease is summarized in [24]. Despite the role of EVs in intra-host cell communication, they have recently garnered attention in mediating inter-kingdom communication as well. In this regard, Lee et al. discuss the results of recent studies that have examined the ways in which EVs and small RNAs mediate “microbe–host” and “host–microbe” interspecies communication [25]. More generally, the impact of commens and bacteria derived-EVs on cell function and their impact on health and disease are summarized as well in [26].

Based on the different methods of EV biogenesis, we hypothesize that exosomes and ectosomes mainly differ in their role in cell–cell communication. In detail, while exosome biogenesis and protein cargo sorting and release are rather complex and time-consuming processes, ectosome shedding happens fast and directly through the outward budding and fission of the plasma membrane and, hence, their surface markers mirror the membrane of origin [27]. Although we have to be aware of impurities in EV subpopulations derived after isolation procedures when assessing the results of studies, the literature points to exosomes as selective carriers of small RNAs, including microRNA [28,29,30,31,32], whereas ectosomes may rather exert their function through the delivery of surface receptors / proteins [10].

With regards to the developments and results to date, we expect a number of exciting scientific projects in the future. Therefore, we would like to interest you in the next Special Issue of the International Journal of Molecular Sciences entitled “The emerging role of EVs in experimental oncology”.
